# Supplementary figures and images for: Transcriptional Response of Candida auris to the Mrr1 Inducers Methylglyoxal and Benomyl
Source: mSphere. 2022 Apr 27;7(3):e00124-22. doi: 10.1128/msphere.00124-22 (PMC9241502; doi:10.1128/msphere.00124-22)

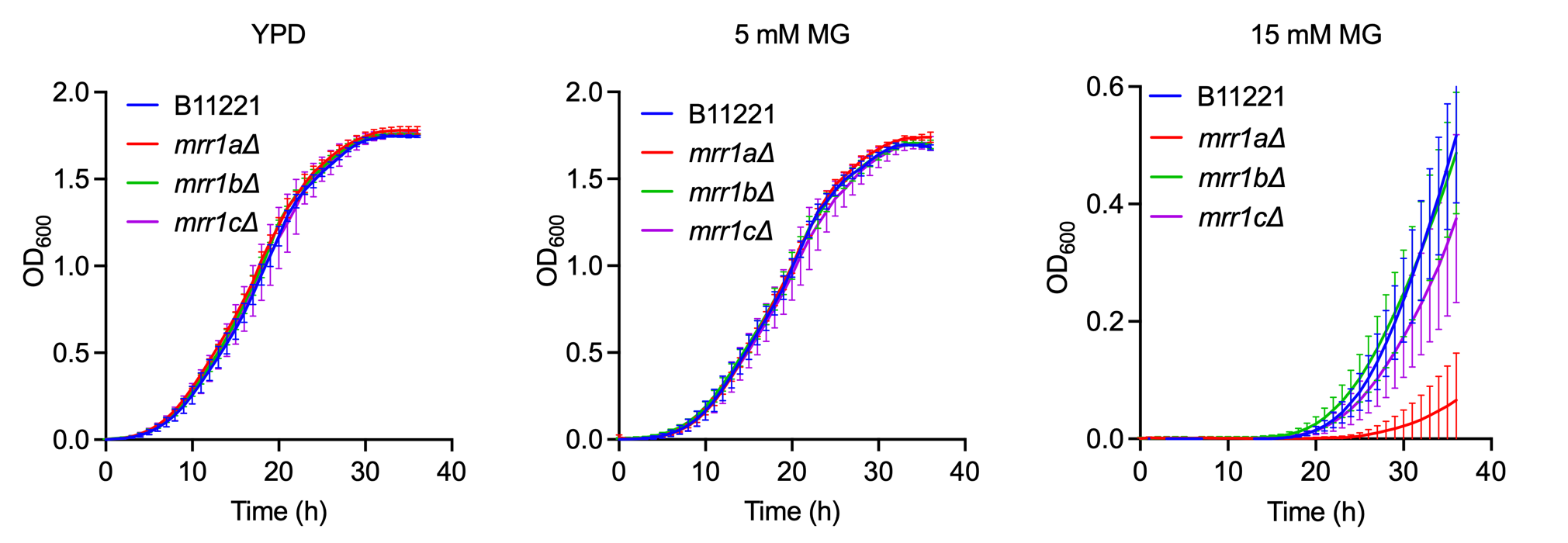

Supplement: FIG S1 [file msphere.00124-22-s0006.tif]

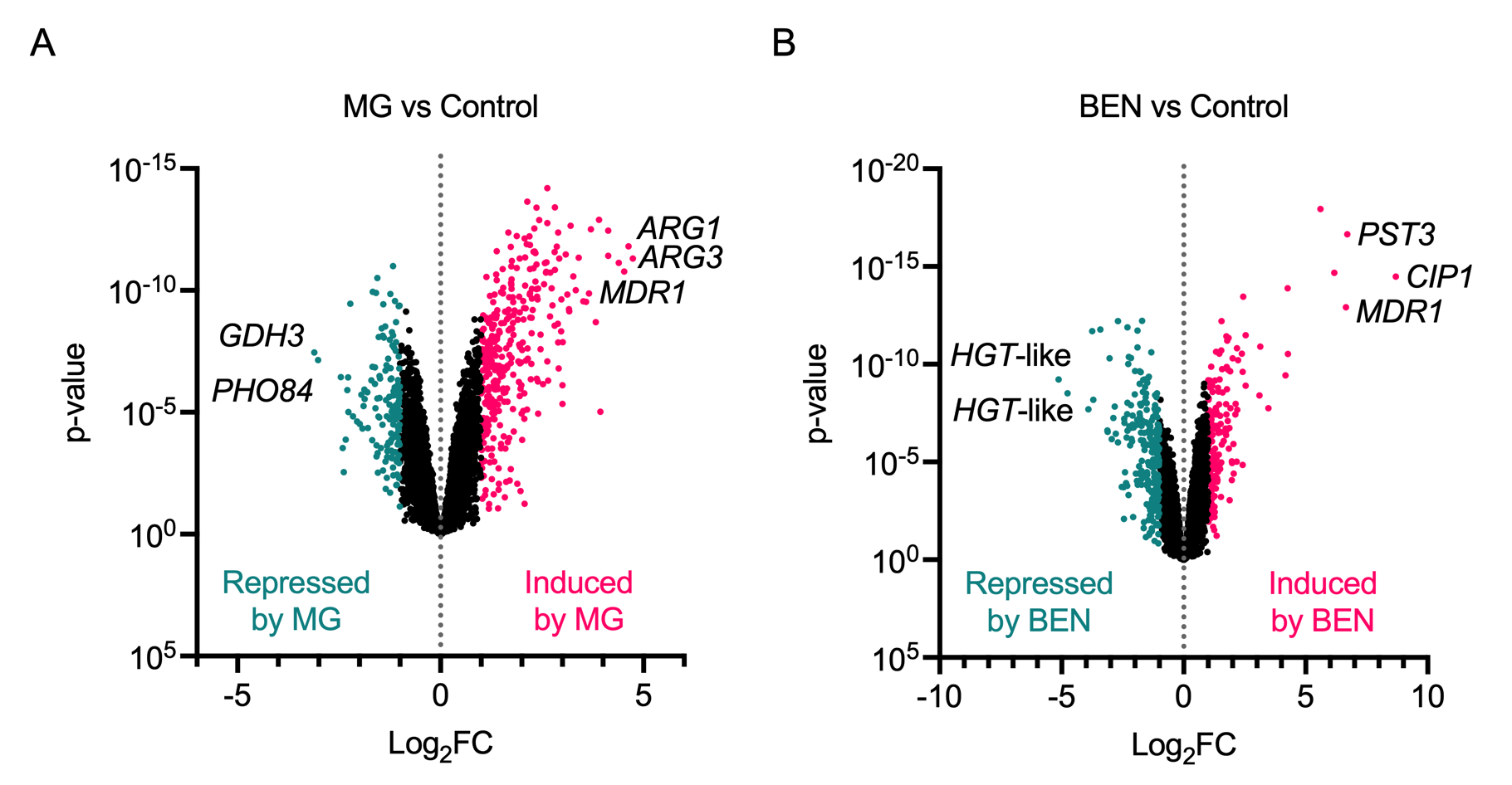

Supplement: FIG S2 [file msphere.00124-22-s0007.tif]

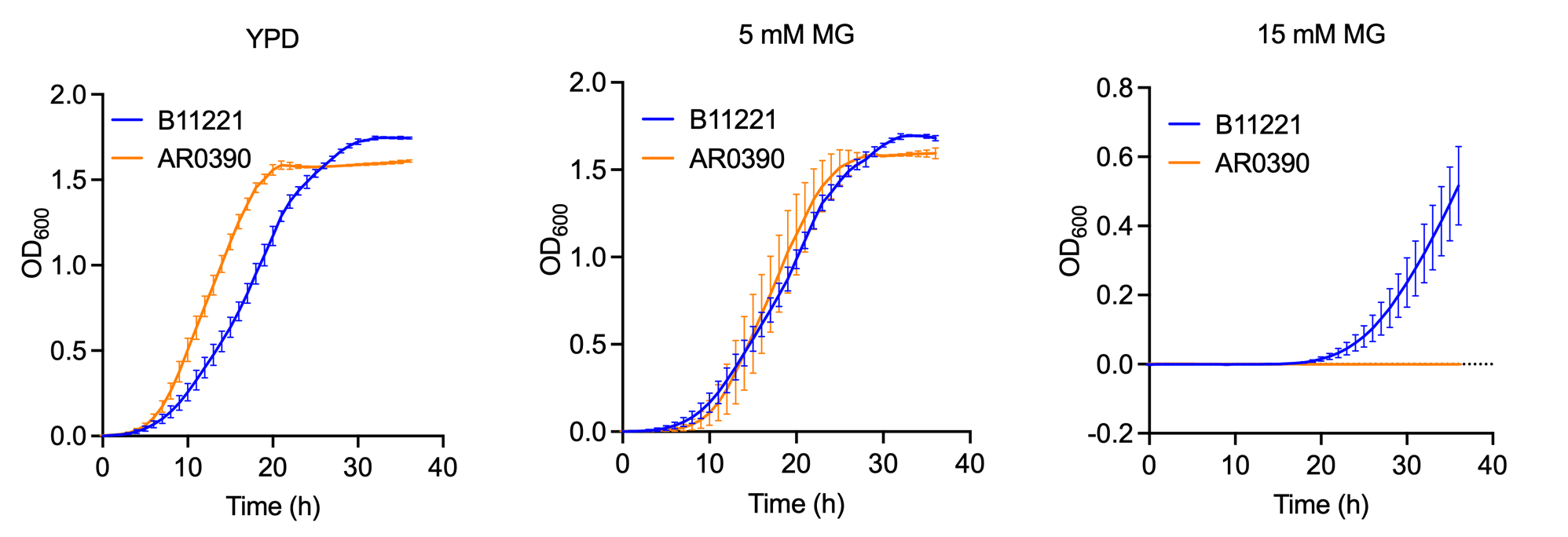

Supplement: FIG S3 [file msphere.00124-22-s0008.tif]

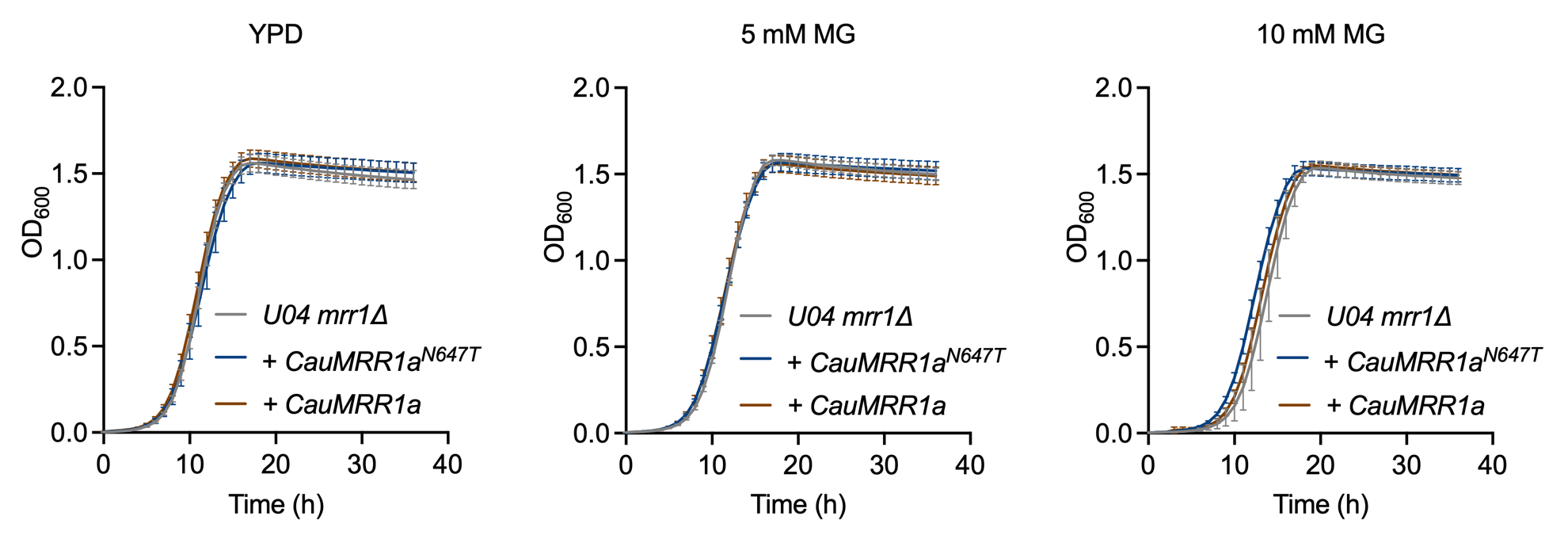

Supplement: FIG S4 [file msphere.00124-22-s0009.tif]

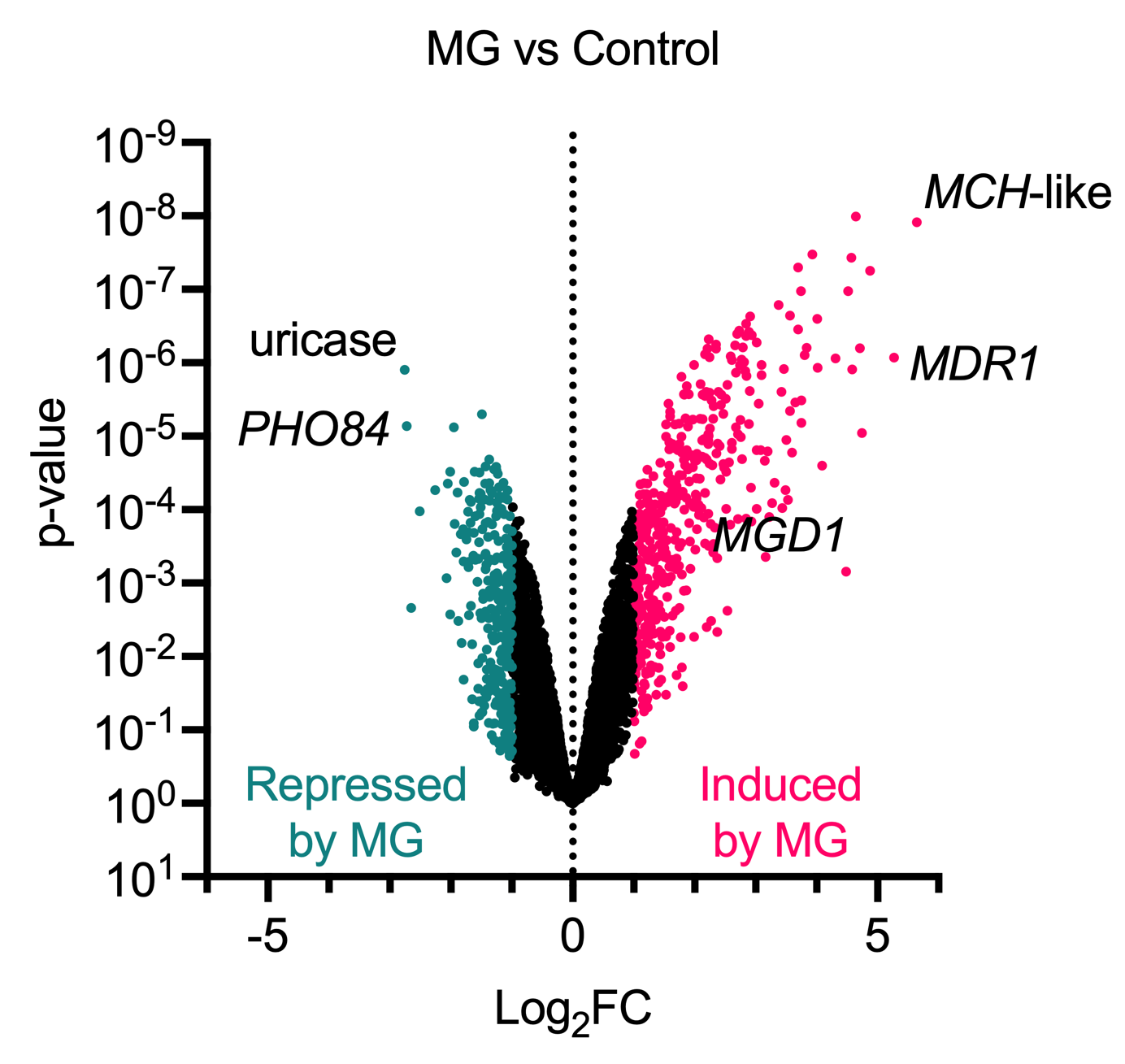

Supplement: FIG S5 [file msphere.00124-22-s0010.tif]
