# Supplementary material for: Transcriptional Response of Candida auris to the Mrr1 Inducers Methylglyoxal and Benomyl
Source: mSphere. 2022 Apr 27;7(3):e00124-22. doi: 10.1128/msphere.00124-22 (PMC9241502; doi:10.1128/msphere.00124-22)
Supplement: TABLE S1 [file msphere.00124-22-s0002.docx]

**Table S1.**

| Sulfur compound assimilation and biosynthesis | | | | | | |
| --- | --- | --- | --- | --- | --- | --- |
| Locus Tag | **Gene Name** | **Predicted function** | **WT MG Log_2_FC** | **WT BEN Log_2_FC** | ***mrr1a*∆ MG Log_2_FC** | ***mrr1a*∆ BEN Log_2_FC** |
| CJI97_001242 | *AGP3* | Serine transporter | 1.06 | 0.80 | 1.36 | 0.42 |
| CJI97_002494 | *DUG1* | Glutathione hydrolase | 1.42 | -0.02 | 1.38 | -0.46 |
| CJI97_001665 | *CYS3* | Peroxisomal cystathionine beta-lyase | 1.37 | 0.42 | 1.39 | 0.30 |
| CJI97_001939 | *CFD1* | Role in Fe-S cluster assembly | -0.02 | 1.17 | 0.26 | 1.31 |
| CJI97_001514 | *CIA1* | Role in protein maturation by Fe-S cluster transfer | 0.40 | 1.12 | 0.34 | 1.20 |
| CJI97_004156 | *DRE2* | Cytosolic Fe-S protein assembly protein | 1.09 | 1.08 | 1.15 | 0.98 |
| CJI97_001503 | *ECM4* | Cytoplasmic glutathione S-transferase | 0.48 | 1.55 | 0.33 | 1.73 |
| CJI97_001382 | *ECM17* | Sulfite reductase beta subunit | 1.14 | 0.76 | 1.45 | 0.33 |
| CJI97_001705 | *GCS1* | Gamma-glutamylcysteine synthetase | 0.78 | 2.00 | 1.06 | 1.75 |
| CJI97_003892 | *GLR1* | Glutathione reductase | -0.04 | 1.17 | 0.06 | 1.11 |
| CJI97_005081 | *GSH2* | Glutathione synthase | 1.07 | 1.41 | 0.98 | 1.13 |
| CJI97_003274 | *GTO1* | Cytoplasmic glutathione S-transferase | 1.39 | 3.93 | 1.93 | 4.27 |
| CJI97_001739 | *JLP1* | Sulfonate dioxygenase | 0.38 | 1.44 | 0.42 | 1.32 |
| CJI97_002076 | *MET1* | Uroporphyrin-3 C-methyltransferase | 1.18 | 1.55 | 1.99 | 1.33 |
| CJI97_002761 | *MET2* | Homoserine acetyltransferase | 2.20 | 0.49 | 2.31 | 0.30 |
| CJI97_004689 | *MET8* | Dehydrogenase, ferrochelatase | 1.83 | 0.30 | 1.65 | 0.65 |
| CJI97_003625 | *MET10* | Sulfite reductase | 1.01 | 0.94 | 1.31 | 0.86 |
| CJI97_003066 | *MET14* | Adenylylsulfate kinase | 1.08 | 0.27 | 1.50 | 0.61 |
| CJI97_005391 | *MET16* | 3'-phosphoadenylsulfate reductase | 1.63 | 1.44 | 1.95 | 1.29 |
| CJI97_003613 | *MUP1* | High affinity methionine permease | 1.05 | 0.36 | 0.94 | -0.06 |
| CJI97_004493 | *MUP3* | L-methionine transmembrane transporter | 0.31 | 1.65 | 0.20 | 1.59 |
| CJI97_004842 | N/A | Role in Fe-S cluster assembly | -0.08 | 1.00 | 0.08 | 0.60 |
| CJI97_005600 | N/A | Cystathionine gamma-synthase | 1.56 | 0.34 | 1.45 | 0.52 |
| CJI97_001635 | N/A | Glutathione S-conjugate transporter | 0.05 | 1.10 | 0.12 | 0.90 |
| CJI97_000433 | *SPE2* | S-adenosylmethionine decarboxylase | 1.11 | 0.19 | 1.14 | 0.05 |
| CJI97_000171 | *SRX1* | Sulfiredoxin | 2.21 | 3.07 | 2.03 | 3.47 |
| CJI97_003300 | *STR2* | Cystathionine gamma-synthase | 1.15 | 0.21 | 1.11 | 0.15 |
| CJI97_001014 | *SUL2* | Sulfate transporter | 1.98 | 1.42 | 3.01 | 1.17 |
| CJI97_003257 | *TES1* | Acyl-CoA thioesterase | 1.27 | 0.59 | 1.06 | 0.38 |
| CJI97_001516 | *TRR1* | Thioredoxin reductase | 0.40 | 1.60 | 0.48 | 1.82 |
| CJI97_000545 | *TRX1* | Thioredoxin | -0.33 | 1.20 | -0.82 | 2.05 |
|  |  |  |  |  |  |  |
| Xenobiotic/Drug Transport | | | | | | |
| Locus Tag | **Gene Name** | **Predicted function** | **WT MG Log_2_FC** | **WT BEN Log_2_FC** | ***mrr1a*∆ MG Log_2_FC** | ***mrr1a*∆ BEN Log_2_FC** |
| CJI97_002597 | *AMF1* | MFS family transporter | 0.35 | 1.28 | 0.71 | 1.29 |
| CJI97_000167 | *CDR1* | ABC family multidrug transporter | 0.30 | 1.88 | 0.40 | 1.76 |
| CJI97_000479 | *CDR4* | ABC family multidrug transporter | 1.34 | 1.13 | 1.27 | 1.14 |
| CJI97_004181 | *ERC1* | Xenobiotic transmembrane transporter | 1.47 | 1.46 | 1.96 | 1.44 |
| CJI97_004982 | *ESBP6* | MFS membrane transporter | 3.97 | 0.66 | 3.90 | 0.20 |
| CJI97_002850 | *FLU1* | Multidrug efflux pump of the plasma membrane | 0.18 | 1.25 | 0.35 | 1.17 |
| CJI97_002639 | *MCH2* | MFS membrane transporter | 1.36 | 0.04 | 1.24 | -0.01 |
| CJI97_000609 | *MCH4* | MFS membrane transporter | 2.13 | 0.47 | 2.14 | 0.33 |
| CJI97_004042 | *MDR1* | Plasma membrane MDR/MFS multidrug efflux protein | 3.83 | 5.60 | 3.65 | 6.65 |
| CJI97_000797 | N/A | MFS membrane transporter | 3.29 | 1.68 | 3.17 | 0.98 |
| CJI97_005702 | N/A | ABC family multidrug transporter | 1.22 | 1.94 | 1.10 | 2.42 |
| CJI97_005706 | N/A | ABC family multidrug transporter | 0.86 | 1.93 | 0.78 | 1.99 |
| CJI97_005256 | *QDR3* | MFS membrane transporter | 2.83 | -1.94 | 2.63 | -2.88 |
| CJI97_005513 | *ROA1* | PDR-subfamily ABC transporter | 2.86 | 0.05 | 3.58 | 0.01 |
| CJI97_001481 | *SNQ2* | ABC family multidrug transporter | 1.50 | 2.56 | 1.34 | 2.19 |
| CJI97_001817 | *VBA1* | MFS transporter | 0.68 | 1.18 | 0.64 | 0.95 |
|  |  |  |  |  |  |  |
| Amino acid biosynthesis, excluding sulfur-containing amino acids | | | | | | |
| Locus Tag | **Gene Name** | **Predicted function** | **WT MG Log_2_FC** | **WT BEN Log_2_FC** | ***mrr1a*∆ MG Log_2_FC** | ***mrr1a*∆ BEN Log_2_FC** |
| CJI97_000687 | *ARG1* | Argininosuccinate synthase | 4.72 | 0.81 | 4.63 | 0.55 |
| CJI97_004654 | *ARG3* | Ornithine carbamoyltransferase | 4.77 | 1.52 | 4.74 | 0.91 |
| CJI97_002308 | *ARG4* | Argininosuccinate lyase | 1.75 | 0.39 | 1.67 | 0.21 |
| CJI97_001846 | *ARG5,6* | Arginine biosynthetic enzyme | 1.50 | 0.60 | 1.46 | 0.19 |
| CJI97_005293 | *ARG8* | Acetylornithine aminotransferase | 1.58 | 0.23 | 1.67 | 0.17 |
| CJI97_002234 | *ARO1* | Pentafunctional arom enzyme | 1.49 | -0.06 | 1.32 | -0.20 |
| CJI97_000465 | *ARO2* | Chorismate synthase | 2.40 | 0.53 | 2.37 | 0.12 |
| CJI97_001597 | *ARO3* | 3-deoxy-D-arabinoheptulosonate-7-phosphate synthase | 1.74 | -0.12 | 1.64 | -0.02 |
| CJI97_003954 | *ARO4* | 3-deoxy-D-arabinoheptulosonate-7-phosphate synthase | 2.50 | 0.36 | 2.38 | 0.18 |
| CJI97_003913 | *ARO7* | Chorismate mutase | 1.51 | 0.08 | 1.30 | 0.37 |
| CJI97_001973 | *ASN1* | Asparagine synthetase | 2.32 | -0.20 | 2.06 | -0.39 |
| CJI97_001997 | *BAT21* | Branched chain amino acid aminotransferase | 2.51 | 0.24 | 2.61 | 0.10 |
| CJI97_002013 | *CPA2* | Carbamoyl-phosphate synthase subunit | 1.78 | 0.15 | 1.83 | -0.37 |
| CJI97_005329 | *HIS1* | ATP phosphoribosyl transferase | 3.40 | 0.97 | 3.27 | 0.57 |
| CJI97_003017 | *HIS3* | Imidazoleglycerol-phosphate dehydratase | 3.20 | 1.31 | 2.97 | 1.46 |
| CJI97_003537 | *HIS4* | Phosphoribosyl-AMP cyclohydrolase, phosphoribosyl-ATP diphosphatase, and histidinol dehydrogenase | 2.86 | 0.94 | 2.73 | 0.33 |
| CJI97_003604 | *HIS5* | Histidinol-phosphate aminotransferase | 2.72 | 0.73 | 2.60 | 0.49 |
| CJI97_003946 | *HIS7* | Imidazole glycerol phosphate synthase | 2.39 | 0.63 | 2.34 | 0.55 |
| CJI97_003721 | *HOM2* | Aspartate-semialdehyde dehydrogenase | 1.52 | 0.23 | 1.40 | 0.20 |
| CJI97_003292 | *HOM3* | L-aspartate 4-P-transferase | 3.04 | 0.61 | 3.08 | 0.35 |
| CJI97_003178 | *ILV1* | Threonine dehydratase | 2.06 | 0.53 | 1.93 | 0.28 |
| CJI97_002682 | *ILV2* | Acetolactate synthase | 2.59 | 0.23 | 2.40 | -0.17 |
| CJI97_000020 | *ILV3* | Dihydroxyacid dehydratase | 2.77 | 0.75 | 2.60 | 0.37 |
| CJI97_003514 | *ILV5* | Ketol-acid reductoisomerase | 1.99 | -0.29 | 2.04 | -0.50 |
| CJI97_004523 | *ILV6* | Regulatory subunit of acetolacetate synthase | 1.50 | 0.30 | 1.25 | 0.31 |
| CJI97_004671 | *LEU1* | 3-isopropylmalate dehydratase | 1.45 | -0.06 | 1.30 | -0.31 |
| CJI97_001329 | *LEU4* | 2-isopropylmalate synthase | 4.41 | 0.44 | 4.13 | -0.12 |
| CJI97_003280 | *LYS1* | Saccharopine dehydrogenase | 2.89 | 0.33 | 2.81 | 0.11 |
| CJI97_003346 | *LYS2* | Alpha-aminoadipate reductase, large subunit | 2.47 | 0.38 | 2.24 | -0.31 |
| CJI97_002417 | *LYS4* | Homoaconitase | 3.08 | 1.06 | 2.92 | 0.50 |
| CJI97_002151 | *LYS5* | Phosphopantetheinyl transferase | 2.70 | 0.24 | 2.55 | 0.29 |
| CJI97_001920 | *LYS9* | Saccharopine dehydrogenase | 1.13 | 0.07 | 0.96 | -0.06 |
| CJI97_003796 | *LYS22* | Homocitrate synthase | 2.91 | 0.07 | 2.82 | -0.33 |
| CJI97_003176 | *SER1* | 3-phosphoserine aminotransferase | 2.11 | 0.16 | 2.05 | 0.15 |
| CJI97_003156 | *SER2* | Phosphoserine phosphatase | 1.29 | 0.14 | 1.34 | 0.34 |
| CJI97_000320 | *SER33* | Enzyme of amino acid biosynthesis | 1.05 | -0.29 | 1.03 | -0.51 |
| CJI97_003863 | *SHM1* | Mitochondrial serine hydroxymethyltransferase | 1.30 | -0.11 | 1.22 | -0.01 |
| CJI97_001157 | *THR1* | Homoserine kinase | 1.82 | 0.41 | 1.75 | 0.25 |
| CJI97_000379 | *TRP2* | Anthranilate synthase | 1.05 | 0.42 | 0.91 | 0.08 |
| CJI97_003979 | *TRP3* | Indole-3-glycerol-phosphate synthase, anthranilate synthase | 1.33 | -0.02 | 1.27 | 0.12 |
| CJI97_003855 | *TRP4* | Enzyme of amino acid biosynthesis | 1.25 | 0.06 | 1.17 | -0.05 |
| CJI97_003424 | *TRP5* | Tryptophan synthase | 2.45 | 0.28 | 2.25 | 0.17 |
|  |  |  |  |  |  |  |
| Redox homeostasis and stress response | | | | | | |
| Locus Tag | **Gene Name** | **Predicted function** | **WT MG Log_2_FC** | **WT BEN Log_2_FC** | ***mrr1a*∆ MG Log_2_FC** | ***mrr1a*∆ BEN Log_2_FC** |
| CJI97_001841 | *CAT1* | Catalase | 0.54 | 2.49 | 0.67 | 2.38 |
| CJI97_003095 | *CIP1* | Oxidoreductase | 0.51 | 8.04 | 1.33 | 8.70 |
| CJI97_002824 | *FDH3* | Oxidoreductase and zinc ion binding activity | -0.61 | 1.14 | 1.22 | 1.22 |
| CJI97_000658 | *MGD1* | NAD(H)-linked methylglyoxal oxidoreductase | 1.35 | 2.46 | -0.46 | 0.66 |
| CJI97_002022 | N/A | Quinone oxidoreductase | 0.36 | 1.98 | 0.53 | 2.18 |
| CJI97_002187 | N/A | Oxidoreductase | -0.04 | 1.07 | -0.07 | 0.97 |
| CJI97_004869 | N/A | Oxidoreductase | 0.33 | 1.58 | 0.16 | 1.57 |
| CJI97_004704 | *POS5* | Mitochondrial NADH kinase | 1.41 | -0.46 | 1.54 | -0.59 |
| CJI97_004613 | *PST3* | Flavodoxin-like protein | 0.41 | 6.37 | 0.38 | 6.71 |
| CJI97_000530 | *SOD2* | Mitochondrial superoxide dismutase | -0.26 | 1.02 | -0.39 | 1.41 |
| CJI97_001282 | *YAH1* | Oxidoreductase | 1.01 | 0.99 | 0.87 | 1.07 |
| CJI97_002560 | *YCF1* | Glutathione S-conjugate transporter | 4.58 | 0.85 | 0.12 | 0.90 |
| CJI97_004612 | *YCP4* | Flavodoxin-like protein | 0.63 | 5.35 | 0.60 | 5.61 |
|  |  |  |  |  |  |  |
| Ergosterol biosynthesis | | | | | | |
| Locus Tag | **Gene Name** | **Predicted function** | **WT MG Log_2_FC** | **WT BEN Log_2_FC** | ***mrr1a*∆ MG Log_2_FC** | ***mrr1a*∆ BEN Log_2_FC** |
| CJI97_000262 | *ERG1* | Squalene epoxidase | -0.85 | -1.43 | -0.68 | -1.38 |
| CJI97_003811 | *ERG3* | C-5 sterol desaturase | -0.96 | -1.11 | -0.70 | -0.73 |
| CJI97_005423 | *ERG6* | Delta(24)-sterol C-methyltransferase | -1.01 | -1.53 | -0.78 | -1.09 |
| CJI97_005634 | *ERG10* | Acetyl-CoA acetyltransferase | -0.93 | -1.04 | -1.07 | -0.77 |
| CJI97_005638 | *ERG10* | Acetyl-CoA acetyltransferase | -1.48 | -0.63 | -0.71 | -1.63 |
| CJI97_001156 | *ERG11* | Lanosterol 14-alpha-demethylase | -0.24 | -1.40 | -0.13 | -1.09 |
|  |  |  |  |  |  |  |
| Metal acquisition, including regulation | | | | | | |
| Locus Tag | **Gene Name** | **Predicted function** | **WT MG Log_2_FC** | **WT BEN Log_2_FC** | ***mrr1a*∆ MG Log_2_FC** | ***mrr1a*∆ BEN Log_2_FC** |
| CJI97_002517 | *CTR1* | Copper transporter | -1.13 | -0.26 | -1.24 | 0.01 |
| CJI97_000015 | *FRE7* | Ferric reductase | -1.23 | -0.11 | -1.43 | -0.40 |
| CJI97_003972 | *FRE8* | Iron/copper reductase | -1.03 | -1.18 | -0.90 | -1.30 |
| CJI97_004532 | *FRP1* | Ferric reductase | -1.41 | -1.39 | -0.85 | -1.66 |
| CJI97_001154 | N/A | Ferric or cupric reductase | -0.57 | -1.79 | -0.97 | -2.16 |
| CJI97_004566 | N/A | Ferric or cupric reductase | -1.43 | -1.46 | -1.51 | -1.81 |
| CJI97_005148 | N/A | Ferric or cupric reductase | -1.56 | -0.18 | -1.81 | -0.50 |
| CJI97_002299 | N/A | High affinity iron transporter for intravacuolar stores of iron | -0.19 | -1.79 | -0.16 | -1.54 |
| CJI97_001085 | N/A | Transporter of ferrochrome siderophores | -0.06 | -1.10 | -0.57 | -1.83 |
| CJI97_001117 | N/A | Transporter of ferrochrome siderophores | -1.90 | -2.52 | -1.67 | -2.30 |
| CJI97_001762 | N/A | Transporter of ferrochrome siderophores | -1.94 | -2.41 | -1.87 | -2.31 |
| CJI97_004100 | N/A | Transporter of ferrochrome siderophores | -0.26 | -1.47 | -0.21 | -1.70 |
| CJI97_004165 | N/A | Transporter of ferrochrome siderophores | 0.58 | -1.14 | 0.83 | -0.62 |
| CJI97_001499 | *SEF1* | Zn2-Cys6 transcription factor, regulates iron uptake | -1.13 | 0.31 | -1.10 | 0.02 |
| CJI97_000010 | *ZRT2* | Zinc transporter | -0.38 | -2.50 | -0.80 | -2.32 |
|  |  |  |  |  |  |  |
| Carbohydrate metabolism and biosynthesis | | | | | | |
| Locus Tag | **Gene Name** | **Predicted function** | **WT MG Log_2_FC** | **WT BEN Log_2_FC** | ***mrr1a*∆ MG Log_2_FC** | ***mrr1a*∆ BEN Log_2_FC** |
| CJI97_003911 | *DAC1* | N-acetylglucosamine-6-phosphate deacetylase | -0.35 | -2.02 | 0.20 | -2.05 |
| CJI97_005247 | *FBP1* | Fructose-1,6-bisphosphatase | -1.33 | -1.11 | -1.06 | -0.96 |
| CJI97_003057 | *GLC3* | 1,4-glucan branching enzyme | -1.05 | -1.86 | -0.95 | -1.62 |
| CJI97_001045 | *GSY1* | Glycogen synthase | -1.17 | -1.40 | -1.10 | -1.25 |
| CJI97_003909 | *HXK1* | N-acetylglucosamine kinase | -0.53 | -1.15 | -0.36 | -1.37 |
| CJI97_005579 | *MAE1* | Mitochondrial malic enzyme | -1.44 | -1.41 | -1.36 | -1.49 |
| CJI97_000695 | *MDH1* | Mitochondrial malate dehydrogenase | -1.23 | -0.73 | -1.09 | -0.51 |
| CJI97_003910 | *NAG1* | Glucosamine-6-phosphate deaminase | -0.28 | -1.76 | 0.03 | -1.42 |
| CJI97_001805 | N/A | Role in beta-1,6 glucan biosynthesis | -1.16 | -0.30 | -0.95 | -0.84 |
| CJI97_000696 | *NTH1* | Neutral trehalase | -1.07 | -1.46 | -1.20 | -1.43 |
| CJI97_002722 | *PCK1* | Phosphoenolpyruvate carboxykinase | -2.03 | -1.92 | -1.68 | -1.77 |
| CJI97_002654 | *PMM1* | Phosphomannomutase | -1.05 | -1.33 | -1.12 | -0.73 |
|  |  |  |  |  |  |  |
| Glucose transport, including regulation | | | | | | |
| Locus Tag | **Gene Name** | **Predicted function** | **WT MG Log_2_FC** | **WT BEN Log_2_FC** | ***mrr1a*∆ MG Log_2_FC** | ***mrr1a*∆ BEN Log_2_FC** |
| CJI97_000584 | *HGT16* | MFS glucose transporter | 1.38 | -1.96 | 1.28 | -2.14 |
| CJI97_002713 | *HGT17* | MFS glucose transporter | 0.76 | -3.05 | -0.60 | -2.46 |
| CJI97_005108/CJI97_005109 | *HGT19* | MFS glucose/myo-inositol transporter | -1.71 | -2.78 | -1.95 | -2.14 |
| CJI97_001793 | N/A | MFS glucose transporter | -2.00 | -6.23 | -2.27 | -4.76 |
| CJI97_001794 | N/A | MFS glucose transporter | 0.85 | -2.17 | -0.21 | -1.41 |
| CJI97_002023 | N/A | MFS glucose transporter | -1.57 | -5.00 | -1.58 | -5.13 |
| CJI97_002024 | N/A | MFS glucose transporter | -2.02 | -2.80 | -1.69 | -2.70 |
| CJI97_002448 | *RGT1* | Transcriptional repressor of glucose transport | -1.26 | -1.50 | -1.29 | -1.56 |
| CJI97_005617 | *SHA3* | Ser/thr kinase involved in glucose transport | -1.14 | -0.65 | -0.87 | -1.09 |
