# Supplementary material for: Transcriptional Response of Candida auris to the Mrr1 Inducers Methylglyoxal and Benomyl
Source: mSphere. 2022 Apr 27;7(3):e00124-22. doi: 10.1128/msphere.00124-22 (PMC9241502; doi:10.1128/msphere.00124-22)
Supplement: TABLE S2 [file msphere.00124-22-s0003.docx]

**Table S2.**

| Genes more highly expressed in B11221 | | | | | | |
| --- | --- | --- | --- | --- | --- | --- |
| B11221 Locus Tag | **AR0390 Locus Tag** | **Gene Name** | **Predicted Function** | **Log_2_FC (B11221 vs AR0390)** | **B11221 average (CPM)** | **AR0390 average (CPM)** |
| CJI97_004624 | B9J08_004828 | *MGD2* | NAD(H)-linked methylglyoxal oxidoreductase | 11.29 | 1657 | 0.66 |
| CJI97_000658 | B9J08_000656 | *MGD1* | NAD(H)-linked methylglyoxal oxidoreductase | 8.53 | 4335 | 11.7 |
| CJI97_004768 | B9J08_004684 | N/A | Role in histone deacetylation | 7.87 | 51.9 | 0.22 |
| CJI97_000946 | B9J08_000928 | *AQY1* | Aquaporin water channel, osmotic shock resistance | 7.33 | 581 | 3.60 |
| CJI97_004767 | B9J08_004685 | N/A | Curved DNA-binding protein | 5.36 | 279 | 6.78 |
| CJI97_003833 | B9J08_003761 | N/A | DNA topoisomerase | 5.31 | 96.8 | 2.43 |
| CJI97_002880 | B9J08_002824 | N/A | DNA replication licensing factor required for pre-replication complex assembly | 4.49 | 316 | 14.1 |
| CJI97_004042 | B9J08_003981 | *MDR1* | Plasma membrane MDR/MFS multidrug efflux pump | 4.42 | 190 | 8.88 |
| CJI97_002740 | B9J08_002688 | *FDH1* | Formate dehydrogenase | 4.33 | 131 | 6.50 |
| CJI97_004770 | B9J08_004682 | *ECM42* | Ornithine acetyltransferase | 4.24 | 99.9 | 5.28 |
|  |  |  |  |  |  |  |
| Genes more highly expressed in AR0390 | | | | | | |
| Locus Tag | **AR0390 Locus Tag** | **Gene Name** | **Predicted Function** | **Log_2_FC (B11221 vs AR0390)** | **B11221 average (CPM)** | **AR0390 average (CPM)** |
| CJI97_001302 | B9J08_001303 | *BDF1* | Essential chromatin-binding bromodomain protein | -10.21 | 0.48 | 563 |
| CJI97_004515 | B9J08_004451 | N/A | ALS family protein | -9.61 | 1.98 | 1546 |
| CJI97_004556 | B9J08_005565 | *PRD1* | Proteinase | -7.29 | 0.14 | 22.6 |
| CJI97_001865 | B9J08_002322 | *BMH1* | Role in morphology | -7.01 | 12.7 | 1639 |
| CJI97_002974 | B9J08_002900 | *RMD9* | Mitochondrial protein with a predicted role in respiratory growth | -6.86 | 6.41 | 745 |
| CJI97_003073 | B9J08_003002 | N/A | Iron permease | -6.51 | 36.8 | 3350 |
| CJI97_002817 | B9J08_002762 | *INO1* | Inositol-1-phosphate synthase | -5.48 | 28.7 | 1281 |
| CJI97_004514 | B9J08_004450 | *THI13* | Thiamin pyrimidine synthase | -4.95 | 2.34 | 72.4 |
| CJI97_004654 | B9J08_004798 | *ARG3* | Ornithine carbamoyltransferase | -4.95 | 0.70 | 21.5 |
| CJI97_000838 | B9J08_000820 | *SAM4* | S-adenosylmethionine-homocysteine methyltransferase | -4.43 | 1.93 | 41.5 |
