# Supplementary material for: Transcriptional Response of Candida auris to the Mrr1 Inducers Methylglyoxal and Benomyl
Source: mSphere. 2022 Apr 27;7(3):e00124-22. doi: 10.1128/msphere.00124-22 (PMC9241502; doi:10.1128/msphere.00124-22)
Supplement: TABLE S3 [file msphere.00124-22-s0004.docx]

**Table S3.**

| B11221 Locus Tag | AR0390 Locus Tag | Gene Name | Predicted Function | B11221 Log_2_FC | AR0390 Log_2_FC |
| --- | --- | --- | --- | --- | --- |
| CJI97_005163 | B9J08_005078 | *AAH1* | Adenine deaminase, purine salvage and nitrogen catabolism | 0.29 | 1.10 |
| CJI97_002719 | B9J08_002666 | *AGP2* | Amino acid permease | 0.45 | 1.16 |
| CJI97_001242 | B9J08_001362 | *AGP3* | Serine transporter; sulfur assimilation | 1.06 | 0.00 |
| CJI97_004654 | B9J08_004798 | *ARG3* | Ornithine carbamoyltransferase | 4.77 | 3.02 |
| CJI97_003828 | B9J08_003754 | *ARG11* | Ornithine transporter of the mitochondrial inner membrane | 0.94 | 1.70 |
| CJI97_003954 | B9J08_003882 | *ARO4* | 3-deoxy-D-arabinoheptulosonate-7-phosphate synthase; aromatic amino acid biosynthesis | 2.50 | 2.22 |
| CJI97_001997 | B9J08_002453 | *BAT21* | Branched chain amino acid aminotransferase | 2.51 | 2.56 |
| CJI97_005185 | B9J08_005101 | *BNA1* | 3-hydroxyanthranilic acid dioxygenase; NAD biosynthesis | 1.54 | -0.33 |
| CJI97_000479 | B9J08_000479 | *CDR4* | ABC transporter superfamily | 1.34 | 1.01 |
| CJI97_003095 | B9J08_003024 | *CIP1* | Oxidoreductase | 0.51 | 1.30 |
| CJI97_004156 | B9J08_004088 | *DRE2* | Cytosolic Fe-S protein assembly protein | 1.09 | 2.52 |
| CJI97_004181 | B9J08_004118 | *ERC1* | Xenobiotic transmembrane transporter | 1.47 | 0.00 |
| CJI97_002824 | B9J08_002769 | *FDH3* | Oxidoreductase and zinc ion binding activity | 1.25 | 0.90 |
| CJI97_005329 | B9J08_005247 | *HIS1* | ATP phosphoribosyl transferase; histidine biosynthesis | 3.40 | 2.90 |
| CJI97_001933 | B9J08_002388 | *HST6* | ABC transporter related to mammalian P-glycoproteins | 0.66 | 1.18 |
| CJI97_003449 | B9J08_003374 | *ICL1* | Isocitrate lyase; glyoxylate cycle enzyme | 0.79 | 1.33 |
| CJI97_000020 | B9J08_000013 | *ILV3* | Dihydroxyacid dehydratase | 2.77 | 2.73 |
| CJI97_004268 | B9J08_004204 | *JEN1* | Lactate transporter | 1.84 | -0.24 |
| CJI97_001329 | B9J08_001277 | *LEU4* | 2-isopropylmalate synthase | 4.41 | 4.31 |
| CJI97_001920 | B9J08_002375 | *LYS9* | Saccharopine dehydrogenase; lysine biosynthesis | 1.13 | 0.52 |
| CJI97_004689 | B9J08_004763 | *MET8* | Bifunctional dehydrogenase and ferrochelatase; siroheme biosynthesis | 1.83 | 0.94 |
| CJI97_003625 | B9J08_003552 | *MET10* | Sulfite reductase; sulfur amino acid metabolism | 1.01 | 0.90 |
| CJI97_000409 | B9J08_000409 | *MET13* | Methionine biosynthesis protein | 0.04 | 1.65 |
| CJI97_003066 | B9J08_002995 | *MET14* | Adenylylsulfate kinase; sulfur metabolism | 1.08 | 0.93 |
| CJI97_005391 | B9J08_005307 | *MET16* | 3'-phosphoadenylsulfate reductase; sulfur amino acid metabolism | 1.63 | 1.75 |
| CJI97_004042 | B9J08_003981 | *MDR1* | Plasma membrane MDR/MFS multidrug efflux protein | 3.83 | 5.27 |
| CJI97_000658 | B9J08_000656 | *MGD1* | NAD(H)-linked methylglyoxal oxidoreductase | 1.35 | 2.04 |
| CJI97_004624 | B9J08_004828 | *MGD2* | NAD(H)-linked methylglyoxal oxidoreductase | -0.06 | 1.60 |
| CJI97_002730 | B9J08_002677 | *MIS12* | Mitochondrial C1-tetrahydrofolate synthase precursor | 1.05 | 0.87 |
| CJI97_004904 | B9J08_004548 | *NAR1* | Cytosolic iron-sulfur protein assembly machinery protein | 0.84 | 2.13 |
| CJI97_003488 | B9J08_003413 | *OPT7* | Oligopeptide transporter, may transport GSH or related compounds | 1.09 | 0.90 |
| CJI97_001481 | B9J08_001125 | *SNQ2* | Putative ABC transporter superfamily | 1.50 | 2.25 |
| CJI97_003300 | B9J08_003225 | *STR2* | Cystathionine gamma-synthase; sulfur compound metabolism | 1.15 | 1.32 |
| CJI97_001014 | B9J08_000995 | *SUL2* | Sulfate transporter | 1.98 | 2.03 |
| CJI97_004677 | B9J08_004775 | *TPO3* | Polyamine transporter, MFS-MDR family | 0.91 | 1.24 |
| CJI97_002495 | B9J08_001834 | *TPO4* | Spermidine transporter | 0.48 | 1.49 |
| CJI97_003424 | B9J08_003349 | *TRP5* | Tryptophan synthase | 2.45 | 2.52 |
| CJI97_002560 | B9J08_001899 | *YCF1* | Glutathione S-conjugate transporter | 4.58 | 4.57 |
| CJI97_005451 | B9J08_005368 | *YDJ1* | Type I HSP40 co-chaperone | 0.25 | 1.20 |
| CJI97_001435 | B9J08_001171 | *ADH5* | Alcohol dehydrogenase | -1.54 | -1.34 |
| CJI97_002591 | B9J08_001930 | *AOX1* | Alternative oxidase, cyanide-resistant respiration | -1.31 | -0.01 |
| CJI97_004799 | B9J08_004653 | *ARP2* | Component of the Arp2/3 complex | -0.79 | -1.11 |
| CJI97_001469 | B9J08_001137 | *ARP3* | Protein with Myo5p-dependent localization to cortical actin patches at hyphal tip | -0.55 | -1.05 |
| CJI97_000089 | B9J08_000084 | *ATP14* | Mitochondrial F1F0 ATP synthase subunit | -0.45 | -1.02 |
| CJI97_002664 | B9J08_002610 | *ATP17* | Mitochondrial ATPase complex subunit | -0.33 | -1.25 |
| CJI97_003777 | B9J08_003702 | *CDG1* | Cysteine dioxygenases, role in conversion of cysteine to sulfite | -2.12 | -2.65 |
| CJI97_004336 | B9J08_004273 | *COX6* | Cytochrome c oxidase | -0.64 | -1.03 |
| CJI97_002184 | B9J08_001993 | *COX11* | Cytochrome oxidase assembly protein | -0.43 | -1.43 |
| CJI97_004817 | B9J08_004635 | *COX19* | Cytochrome c oxidase assembly protein | -0.39 | -1.16 |
| CJI97_002517 | B9J08_001856 | *CTR1* | Copper transporter | -1.13 | -2.07 |
| CJI97_005423 | B9J08_005340 | *ERG6* | Delta(24)-sterol C-methyltransferase, converts zymosterol to fecosterol, ergosterol biosynthesis | -1.01 | -0.46 |
| CJI97_005321 | B9J08_005239 | *FBA1* | Fructose-bisphosphate aldolase | -0.72 | -1.13 |
| CJI97_005247 | B9J08_005163 | *FBP1* | Fructose-1,6-bisphosphatase, key gluconeogenesis enzyme | -1.33 | -1.48 |
| CJI97_000015 | B9J08_000008 | *FRE7* | Ferric reductase | -1.23 | -0.86 |
| CJI97_003972 | B9J08_004052 | *FRE8* | Iron/copper reductase | -1.03 | -0.23 |
| CJI97_004532 | B9J08_004468 | *FRP1* | Ferric reductase | -1.41 | -0.53 |
| CJI97_002942 | B9J08_002886 | *GIT3* | Glycerophosphocholine permease | -1.18 | -0.13 |
| CJI97_003057 | B9J08_002986 | *GLC3* | 1,4-glucan branching enzyme | -1.05 | -0.93 |
| CJI97_004438 | B9J08_004375 | *GPM1* | Phosphoglycerate mutase | -0.53 | -1.40 |
| CJI97_001045 | B9J08_001025 | *GSY1* | Glycogen synthase | -1.17 | -1.36 |
| CJI97_005108/ CJI97_005109 | B9J08_005025 | *HGT19* | MFS glucose/myo-inositol transporter | -1.71 | -0.98 |
| CJI97_002699 | B9J08_002646 | *HXT5* | Sugar transporter | 0.13 | -1.13 |
| CJI97_002817 | B9J08_002762 | *INO1* | Inositol-1-phosphate synthase | -0.36 | -1.41 |
| CJI97_001658 | B9J08_001652 | *JAC1* | ATPase activator activity | -0.25 | -1.04 |
| CJI97_005579 | B9J08_005497 | *MAE1* | Mitochondrial malic enzyme | -1.44 | -1.10 |
| CJI97_000695 | B9J08_000694 | *MDH1* | Mitochondrial malate dehydrogenase | -1.23 | -1.26 |
| CJI97_002683 | B9J08_002630 | *MEP1* | Ammonium permease | -1.81 | -1.38 |
| CJI97_003663 | B9J08_003590 | *MIG2* | Transcription factor involved in glucose repression | -1.17 | -1.44 |
| CJI97_002101 | B9J08_002556 | *MIX14* | Role in aerobic respiration and mitochondrial intermembrane space localization | -0.20 | -1.38 |
| CJI97_002993 | B9J08_002919 | *MLS1* | Malate synthase, glyoxylate cycle enzyme | -0.84 | -1.11 |
| CJI97_001141 | B9J08_001463 | *MYO1* | Component of actomyosin ring at neck of newly emerged bud | -0.98 | -1.22 |
| CJI97_001117 | B9J08_001487 | N/A | Transporter of ferrochrome siderophores | -1.90 | -1.06 |
| CJI97_001762 | B9J08_001547 | N/A | Transporter of ferrochrome siderophores | -1.94 | -1.07 |
| CJI97_000596 | B9J08_000675 | N/A | Adhesin-like protein | -1.12 | 0.15 |
| CJI97_002126 | B9J08_002582 | N/A | Adhesin-like protein | -1.48 | 1.00 |
| CJI97_003987 | B9J08_004037 | N/A | Adhesin-like protein | -1.19 | -0.35 |
| CJI97_004240 | B9J08_004176 | N/A | Secreted lipase | -1.69 | -0.78 |
| CJI97_001776 | B9J08_001533 | N/A | NAD-aldehyde dehydrogenase | -1.57 | -1.55 |
| CJI97_003161 | B9J08_003088 | N/A | NAD-aldehyde dehydrogenase | -1.76 | -1.89 |
| CJI97_001793 | B9J08_002250 | N/A | MFS glucose transporter | -2.00 | -1.41 |
| CJI97_002024 | B9J08_002481 | N/A | MFS glucose transporter | -2.02 | -1.52 |
| CJI97_004566 | B9J08_004886 | N/A | Protein similar to ferric reductases and cupric reductases | -1.43 | -1.43 |
| CJI97_005148 | B9J08_005064 | N/A | Protein similar to ferric reductases and cupric reductases | -1.56 | -1.84 |
| CJI97_000696 | B9J08_000695 | *NTH1* | Neutral trehalase | -1.07 | -0.98 |
| CJI97_002722 | B9J08_002669 | *PCK1* | Phosphoenolpyruvate carboxykinase | -2.03 | -2.01 |
| CJI97_002521 | B9J08_001860 | *PGK1* | Phosphoglycerate kinase | -0.77 | -1.19 |
| CJI97_001140 | B9J08_001464 | *PHO84* | High-affinity phosphate transporter | -2.74 | -2.72 |
| CJI97_001580 | B9J08_002202 | *PHO89* | Phosphate permease | -1.48 | -0.73 |
| CJI97_001697 | B9J08_001613 | *PHO100* | Putative inducible acid phosphatase | -1.38 | -1.60 |
| CJI97_004666 | B9J08_004786 | *PIR1* | 1,3-beta-glucan-linked cell wall protein | -0.44 | -1.12 |
| CJI97_002654 | B9J08_002600 | *PMM1* | Phosphomannomutase, enzyme of O- and N-linked mannosylation | -1.05 | -0.94 |
| CJI97_002321 | B9J08_002130 | *PUT1* | Putative proline oxidase | -2.49 | -1.79 |
| CJI97_004379 | B9J08_004317 | *PUT2* | Putative delta-1-pyrroline-5-carboxylate dehydrogenase | -1.01 | -1.45 |
| CJI97_002448 | B9J08_001787 | *RGT1* | Transcriptional repressor involved in the regulation of glucose transporter genes | -1.26 | -1.62 |
| CJI97_002974 | B9J08_002900 | *RMD9* | Mitochondrial protein with a predicted role in respiratory growth | -0.25 | -1.09 |
| CJI97_004415 | B9J08_004352 | *SAH1* | S-adenosyl-L-homocysteine hydrolase | -1.46 | -1.22 |
| CJI97_004940 | B9J08_004512 | *SCO1* | Copper transporter | -0.79 | -1.06 |
| CJI97_001499 | B9J08_001107 | *SEF1* | Zn2-Cys6 transcription factor, regulates iron uptake | -1.13 | -0.81 |
| CJI97_005617 | B9J08_005567 | *SHA3* | Ser/thr kinase involved in glucose transport | -1.14 | 0.10 |
| CJI97_002536 | B9J08_001875 | *TPI1* | Triose-phosphate isomerase | -0.36 | -1.42 |
| CJI97_003198 | B9J08_003126 | *QCR8* | Ubiquinol cytochrome c reductase | -0.22 | -1.12 |
| CJI97_002481 | B9J08_001820 | *QCR10* | Ubiquinol-cytochrome-c reductase | -0.31 | -1.01 |
| CJI97_003997 | B9J08_004027 | *WOR1* | Transcription factor of white-opaque phenotypic switching in *C. albicans* | -1.22 | -0.33 |
