# Supplementary material for: Transcriptional Response of Candida auris to the Mrr1 Inducers Methylglyoxal and Benomyl
Source: mSphere. 2022 Apr 27;7(3):e00124-22. doi: 10.1128/msphere.00124-22 (PMC9241502; doi:10.1128/msphere.00124-22)
Supplement: TABLE S4 [file msphere.00124-22-s0005.docx]

**Table S4.**

| Fungal Strains | | | | | |
| --- | --- | --- | --- | --- | --- |
| Strain | **Lab #** | **Species** | **Parent** | **Relevant Characteristics**  **(FLZ MIC, µg/mL)** | **Source** |
| AR0390 | DH2777 | *C. auris* |  | Clinical isolate, clade I | (1) |
| B11221 | DH3880 | *C. auris* |  | Clinical isolate, clade III | (2) |
| *mrr1a*∆ | DH3881 | *C. auris* | B11221 | *mrr1a*∆::*caSAT1* | (2) |
| *mrr1b*∆ | DH3882 | *C. auris* | B11221 | *mrr1b*∆::*caSAT1* | (2) |
| *mrr1c*∆ | DH3883 | *C. auris* | B11221 | *mrr1c*∆::*caSAT1* | (2) |
| U04 *mrr1*∆ | DH3306 | *C. lusitaniae* | U04 | *mrr1*Δ::*NAT1*  (4 µg/mL) | (3) |
| U04 *mrr1*∆ + *CauMRR1a^N647T^* clone #1 | DH3914 | *C. lusitaniae* | U04 *mrr1*∆ | *CauMRR1a^N647T^-HygB*  (16 µg/mL) | This study |
| U04 *mrr1*∆ + *CauMRR1a^N647T^* clone #2 | DH3915 | *C. lusitaniae* | U04 *mrr1*∆ | *CauMRR1a^N647T^-HygB*  (16 µg/mL) | This study |
| U04 *mrr1*∆ + *CauMRR1a^N647T^* clone #8 | DH3916 | *C. lusitaniae* | U04 *mrr1*∆ | *CauMRR1a^N647T^-HygB*  (16 µg/mL) | This study |
| U04 *mrr1*∆ + *CauMRR1a* clone #4 | DH3917 | *C. lusitaniae* | U04 *mrr1*∆ | *CauMRR1a-HygB*  (4 µg/mL) | This study |
| U04 *mrr1*∆ + *CauMRR1a* clone #5 | DH3918 | *C. lusitaniae* | U04 *mrr1*∆ | *CauMRR1a-HygB*  (4 µg/mL) | This study |
| U04 *mrr1*∆ + *CauMRR1a* clone #7 | DH3919 | *C. lusitaniae* | U04 *mrr1*∆ | *CauMRR1a-HygB*  (4 µg/mL) | This study |
|  | | | | | |
| Plasmids in *E. coli* (DH5⍺) | | | | | |
| Strain | **Lab #** | **Species** | **Relevant Characteristics**  **(FLZ MIC, µg/mL)** | | **Source** |
| pMQ30*^MRR1-L1191H+Q1197*^* | DH3829 | *E. coli* | *MRR1^L1191H+Q1197*^-HygB* complementation, Gent^R^ | | (5) |
| pMQ30*^CauMRR1aN647T^* | DH3912 | *E. coli* | *CauMRR1a^N647T^-HygB* complementation, Gent^R^ | | This study |
| pMQ30*^CauMRR1a^* | DH3913 | *E. coli* | *CauMRR1a-HygB* complementation, Gent^R^ | | This study |
|  |  |  |  |  |  |
| Primers | | | | | |
| Name | **Description** | | | **Sequence** | **Source** |
| ED222 | *C. auris* *ACT1* qRT Fwd | | \| 5’ – GAA GGA GAT CAC TGC TTT AGC C – 3’ \| \| --- \| | | This study |
| ED223 | *C. auris* *ACT1* qRT Rev | | 5’ – GAG CCA CCA ATC CAC ACA G – 3’ | | This study |
| ED224 | *C. auris* *MDR1* qRT Fwd | | 5’ – GAA GTA TGA TGG CGG GTG – 3’ | | This study |
| ED225 | *C. auris* *MDR1* qRT Rev | | 5’ – CCC AAG AGA GAC GAG CCC – 3’ | | This study |
| AB126 | *C. auris* *MGD1* qRT Fwd | | 5’ – TTC CCC TGA AAT GGA TTT GA – 3’ | | This study |
| AB127 | *C. auris* *MGD1* qRT Rev | | 5’ – GTC TTG GAG CCA TAG TAA CC – 3’ | | This study |
| AB130 | Amplify *C. auris* *MRR1a* for heterologous complementation, Fwd | | 5’ – CTT CAA CTC CGC AAC ACC TGG AAA CTT CAT TAC TAA AGA TGA TGG TAT CTT CGA AAG ATC – 3’ | | This study |
| AB131 | Amplify *C. auris* *MRR1a* for heterologous complementation, Rev | | 5’ – CTT TAC CAG TAA AGT ATC CTT GCC AAA TTT CGT TCC ATA ATT ACA CAT CAA GCA TCT CTT C – 3’ | | This study |
| ED125 | Forward upstream of *C. lusitaniae* *MRR1* to validate complements | | 5’ – GAA AAA GAA GCC AGC AGA CC – 3’ | | (5) |
| ED126 | Reverse upstream of *C. lusitaniae* *MRR1* to validate complements | | 5’ – GGG TAA AGC CAT TGC AGA C – 3’ | | (5) |
| *ACT1-*F | *C. lusitaniae* *ACT1* qRT Fwd | | 5’ – GTA TCG CTG AGC GTA TGC AA – 3’ | | (6) |
| *ACT1*-R | *C. lusitaniae* *ACT1* qRT Rev | | 5’ – GAT GGA TGG TCC AGA CTC GT – 3’ | | (6) |
| ED058 | *C. lusitaniae* *MDR1* qRT Fwd | | 5’ – TCC ATC CAT GGG TCC ATT ATT C – 3’ | | (3) |
| ED059 | *C. lusitaniae* *MDR1* qRT Rev | | 5’ – CTC AAC ACA AGG AAA GCA CAT C – 3’ | | (3) |
| AB039 | *C. lusitaniae* *MGD1* qRT Fwd | | 5' – CGC AGA AAT CCC TAA AGT AAA T – 3' | | (5) |
| AB040 | *C. lusitaniae MGD1* qRT Rev | | 5' – TAC CCT TTG CTT CGT TCT T – 3' | | (5) |
|  |  | |  | |  |
| Other Oligonucleotides | | | | | |
| Name | **Description** | | **Sequence** | | **Source** |
| *NAT1* crRNA | crRNA targeting *NAT1*; used to complement *C. auris MRR1a* alleles into *C. lusitaniae mrr1∆::NAT1* mutant | | 5’ – GGG AAA ACC TTA GTC AAT GG – 3’ | | (5) |

**References**

1. Pathirana RU, Friedman J, Norris HL, Salvatori O, McCall AD, Kay J, Edgerton M. 2018. Fluconazole-Resistant *Candida auris* Is Susceptible to Salivary Histatin 5 Killing and to Intrinsic Host Defenses. Antimicrob Agents Chemother 62.

2. Mayr EM, Ramirez-Zavala B, Kruger I, Morschhauser J. 2020. A Zinc Cluster Transcription Factor Contributes to the Intrinsic Fluconazole Resistance of *Candida auris*. mSphere 5.

3. Demers EG, Biermann AR, Masonjones S, Crocker AW, Ashare A, Stajich JE, Hogan DA. 2018. Evolution of drug resistance in an antifungal-naive chronic *Candida lusitaniae* infection. Proc Natl Acad Sci U S A 115:12040-12045.

4. Demers EG, Stajich JE, Ashare A, Occhipinti P, Hogan DA. 2021. Balancing Positive and Negative Selection: *In Vivo* Evolution of *Candida lusitaniae* *MRR1*. mBio 12.

5. Biermann AR, Demers EG, Hogan DA. 2021. Mrr1 regulation of methylglyoxal catabolism and methylglyoxal-induced fluconazole resistance in *Candida lusitaniae*. Mol Microbiol 115:116-130.

6. Asner SA, Giulieri S, Diezi M, Marchetti O, Sanglard D. 2015. Acquired Multidrug Antifungal Resistance in *Candida lusitaniae* during Therapy. Antimicrob Agents Chemother 59:7715-22.
